# Descending from trees: a Cretaceous winged ice-crawler illuminates the ecological shift and origin of Grylloblattidae

**DOI:** 10.1098/rspb.2025.0557

**Published:** 2025-06-18

**Authors:** Ancheng Peng, Michael S. Engel, Mathieu Boderau, Frédéric Legendre, Yu Liu, Thet Tin Nyunt, Bo Wang, André Nel

**Affiliations:** ^1^ State Key Laboratory of Palaeobiology and Stratigraphy, Nanjing Institute of Geology and Palaeontology, Chinese Academy of Sciences, Nanjing 210008, People’s Republic of China; ^2^ Institut de Systématique, Evolution, Biodiversité (UMR 7205), MNHN, CNRS, SU, EPHE-PSL, UA, CP50, 57 Rue Cuvier, Paris 75005, France; ^3^ Yunnan Key Laboratory for Palaeobiology, Yunnan University, Kunming, Yunnan 650092, People’s Republic of China; ^4^ Division of Invertebrate Zoology, American Museum of Natural History, New York, NY 10024-5192, USA; ^5^ Museum at Prairiefire, Overland Park, KS 66223, USA; ^6^ MEC International Joint Laboratory for Palaeobiology and Palaeoenvironment, Yunnan University, Kunming, Yunnan 650092, People’s Republic of China; ^7^ Southwest United Graduate School, Jiabing Tian Education Academy, Yunnan Normal University, Kunming, Yunnan 650092, People’s Republic of China; ^8^ Department of Geological Survey and Mineral Exploration, Myanma Gems Museum, Ministry of Natural Resources and Environmental Conservation, Nay pyi Taw, Myanmar

**Keywords:** Insecta, Grylloblattodea, morphology, phylogeny, synapomorphy, male genitalia

## Abstract

Extant ice-crawlers (Notoptera: Grylloblattidae) are wingless, ground-dwelling, relict, polyneopteran insects that live in Holarctic cold environments. Their closest living relatives are the similarly apterous bush-crawlers (Notoptera: Mantophasmatodea) from southern Africa, forming together a disjunct bipolar distribution. Meanwhile, numerous winged fossil insects have been assigned to Grylloblattodea, though the lack of defining synapomorphies has complicated efforts to clarify the evolutionary relationships between these fossils and modern wingless ice-crawlers. Here, we report a well preserved winged ice-crawler, *Zygogrylloblatta longipalpa* gen. et sp. nov., from the Albian/Cenomanian of northern Myanmar (*ca* 99 Ma). *Zygogrylloblatta* has the typical forewing venation of Mesozoic ‘stem-Grylloblattodea’, but also exhibits a unique unambiguous synapomorphy of extant Grylloblattidae in male genitalia (coxae IX with apical styli), making it the only fossil accurately related to crown-group Grylloblattidae. In contrast to ground-dwelling habits of extant ice-crawlers, *Zygogrylloblatta* has well developed wings, arolia and true foot pads, supporting a specialized arboreal lifestyle during the mid-Cretaceous. We demonstrate that Grylloblattidae diverged from some winged, arboreal ancestors prior to the mid-Cretaceous, bridging the gap between ancient stem-group and extant Grylloblattidae. Our results reveal previously unknown ecological and morphological diversity in early ice-crawlers and highlight the significance of transitional fossils in tracing the origin of this enigmatic insect lineage.

## 1. Introduction

Extant ice-crawlers or Grylloblattidae Walker, 1932 [1] (Notoptera: Grylloblattodea) are small apterous insects living in cold environments in northwestern North America, Far Eastern Siberia, northern China, Korea and Japan [2,3]. These cryptic insects are currently considered the sister group of Mantophasmatodea Zompro, Klass, Kristensen, Adis, 2002 [4] (bush-crawlers), which are also apterous but live in warm and arid environments in southern Africa [5,6]. Both groups together have a relict, bipolar distribution: ice-crawlers across the North, bush-crawlers in a restricted area of the South.

Extant ice-crawlers are well known for their complete lack of wings, pentamerous tarsi, asymmetrical male genitalia and narrow microhabitats, with derived specializations for living in harsh conditions (cold and high-elevation areas) in which many other insects would perish [2,3,7]. With only *ca* 40 extant species in six genera [8–10], extant ice-crawlers are considered relict, with extinction having greatly reduced their past diversity [3,7,11]. The ensuing problem is that the lineage appears to have been much more diverse and geographically distributed in deep time than it is today. However, no clear synapomorphy supports a clade comprising both extant ice-crawlers and the vast number of extinct winged taxa that are currently attributed to this clade. On one hand, with the continuous effort of morphological and molecular phylogenetic studies, there is now a consensus that extant Grylloblattodea and Mantophasmatodea are a tightly adjoined and well delimited clade [6,12], known as Notoptera Crampton, 1915 [13] (= Xenonomia Terry and Whiting, 2005, is a junior synonym). On the other hand, there is a century-long discussion against the taxonomic attribution of fossil Grylloblattodea.

Initially, many fossil taxa that are now in the Grylloblattodea were put in the so-called Protorthoptera Handlirsch, 1906 [14]. Rasnitsyn [15] was the first to propose that the Mesozoic family Blattogryllidae Rasnitsyn, 1976 [15] (winged) were direct relatives of extant Grylloblattidae owing to morphological similarities in the head, legs and abdomen (sharing ‘a multitude of general morphological features: the structure of the head and its appendages, the form of the leg segments (especially the tarsi), and the form of the abdomen and its appendages’ (English translation of [15, p. 502]). But these characters are vague and there are no clear synapomorphies. Subsequently, many Palaeozoic and Mesozoic winged insects were classified within Grylloblattodea, but the monophyly of this group remains unclear [16] and the situation seemed to move taxa from the problematic catch-all Protorthoptera and place them in an equally problematic catch-all Grylloblattodea s.l. Grylloblattodea were later placed within a putative supraordinal clade, Eoblattida Handlirsch, 1906 [17,18], or Notopterodea Arillo and Engel, 2006 [19], the latter excluding Eoblattidae Handlirsch, 1906 [19].

Morphological evidence suggests that Eoblattidae belong to the Archaeorthoptera [20] and are unrelated to extant Grylloblattidae [21]. Proposed synapomorphies for Grylloblattidae include an unpaired extrusible membranous vesicle between sternites I and II and vestigial euplantulae, but these are either behaviourally convergent or insufficiently known in fossils [22]. Moreover, the presence of coxae IX with apical styli in the male terminalia is a unique unambiguous synapomorphy for Grylloblattidae [23]. A clade containing both Jurassic winged Blattogryllidae and extant Grylloblattidae has been supported by characters such as a head as broad as the pronotum and asymmetrical male gonocoxites; however, these characters are either insufficient or poorly documented in fossils [23], and the former is a poor character given that there is a continuous spectrum of head widths (i.e. extremely labile, with head width varying dramatically across species within an insect family). The asymmetry of gonocoxites of published specimens is particularly challenging to determine owing to limited photographic resolution and specimen availability [24]. Furthermore, a recent morphological phylogenetic analysis based on the study of a winged female specimen of *Aristovia daniili* Storozhenko & Gröhn, 2023 [8] concluded that the winged fossil Grylloblattodea (under the superfluous name ‘Blattogryllopterida’) are related to extant Grylloblattidae, sharing two putative synapomorphies in the lacinia and the galea [25]. As for Mantophasmatodea, the complete loss of wings has been suggested as a potential synapomorphy linking Grylloblattidae with Mantophasmatodea, but excluding fossil winged forms from the extant grylloblattodean stem group [22]. In summary, while a number of potential synapomorphies have been proposed, the lack of definitive fossil evidence has resulted in the relationship between fossil taxa currently in Grylloblattodea and extant Grylloblattidae remaining unclear.

Another remarkable trait of extant ice-crawlers is their constrained thermal breadth and foraging behaviour, which reflects their unique ecological niche and survival strategies [7,11]. Unlike other polyneopteran insects, extant ice-crawlers possess specializations for extreme cold despite being poikilotherms like other insects [11,26–28]. Throughout their evolutionary history, they have undergone significant and irreversible ecological-niche shifts, resulting in evolutionary trade-offs [7,11,27,28]. Specifically, they were subject to continental drift and climate change from the late Palaeogene to the Middle and Late Cretaceous (30–115 Ma) [27], which led to their diversification and shift to underground life, inhabiting cold and humid environments and repeatedly being trapped in a narrow range of habitats [28–31]. These cold environments have imposed on ice-crawlers concessions in energy metabolism control and survival at low temperatures, ultimately limiting the evolution of their thermal niche [32–34]. Correspondingly, extant ice-crawlers have undergone significant morphological changes: they have lost their wings [35], reduced their body size [30,35], and altered their leg structure. In addition, they have evolved physiological mechanisms associated with survival under hard, cold extremes [11]. However, molecular evidence is insufficient to assess the timing of the divergence of their ecological niches [7,11,28,31]. Therefore, clarifying the classification of fossils within Grylloblattodea is crucial for elucidating this important morphological change and shift in their ecological niche. Nevertheless, well preserved fossils of winged ice-crawlers are rare. Only two adults and a nymphal fossil have been found in the mid-Cretaceous Kachin amber and mid-Jurassic Daohugou deposits [8,25,36,37], emphasizing the need for further palaeontological exploration.

Herein, we explore the systematics of Notoptera and particularly Grylloblattodea and the relationships between this group and other polyneopteran orders, and test the putative apomorphies of and within the clade. We take advantage of a newly described, well preserved winged male fossil from mid-Cretaceous Kachin amber that is attributable to Grylloblattodea based on its forewing venation and general habitus. The new fossil represents the latest record of Grylloblattodea fossil occurrences, expanding the current Grylloblattodea’s fossil record to three species and suggesting that the group was diverse during the mid-Cretaceous. Importantly, the male genitalia, studied through three-dimensional rendering and wing venation characters provide evidence for unifying the systematics of extant and fossil groups. This fossil is the first representative of this group, and considering the evolutionary history of living ice-crawlers, this transitional specimen offers insights into their evolutionary path. Additionally, the preservation of distinctive leg characters enables us to compare this fossil with other typical arboreal polyneopteran taxa as well as extant ice-crawlers and bush-crawlers, allowing us to explore shifts in ecological niches through time. In sum, we provide a clearer understanding of the relationships between extant ice-crawlers and their closest winged fossil species, presenting a new perspective on their adaptive evolution in response to environmental changes.

## 2. Results

### (a) Systematic palaeontology

Order Notoptera

Suborder Grylloblattodea

*Remarks.* See electronic supplementary material, data S5 for the complete remarks.

Family Zygogrylloblattidae fam. nov.

Type genus: *Zygogrylloblatta* gen. nov.

*Diagnosis*. As for the type genus (below).

*ZooBank LSID*: urn:lsid:zoobank.org:act:FE69ACA1-D03C−4CE4-B323−9468EAEAEDF7

Genus *Zygogrylloblatta* gen. nov.

Type species: *Zygogrylloblatta longipalpa* sp. nov.

*Etymology*. The new generic name is a combination of the Ancient Greek noun *ζῠγόν*/*zugón*, meaning, ‘yoke’ (in the sense of joining beasts of burden), and the generic name *Grylloblatta* Walker. The gender of the name is feminine.

*Diagnosis*. Maxillary palpus extremely long (a putative autapomorphy); tarsal formula 4−5−5; in forewings, no ‘false costa’; RP with three short, apical branches; M and CuA separated; no ‘arculus’; a broad area between CuA and CuP; basal stem of CuA nearly straight; CuA1 with only two branches; CuA2 forming a strong basal curve; ScP short, only reaching two-third of wing length; only rather few simple crossveins between main veins.

*ZooBank LSID*: urn:lsid:zoobank.org:act:EF1D8AFB−0012−4FC0-AA4B−010609B36941

*Zygogrylloblatta longipalpa* sp. nov.

(figures 1 and 2)

**Figure 1.**
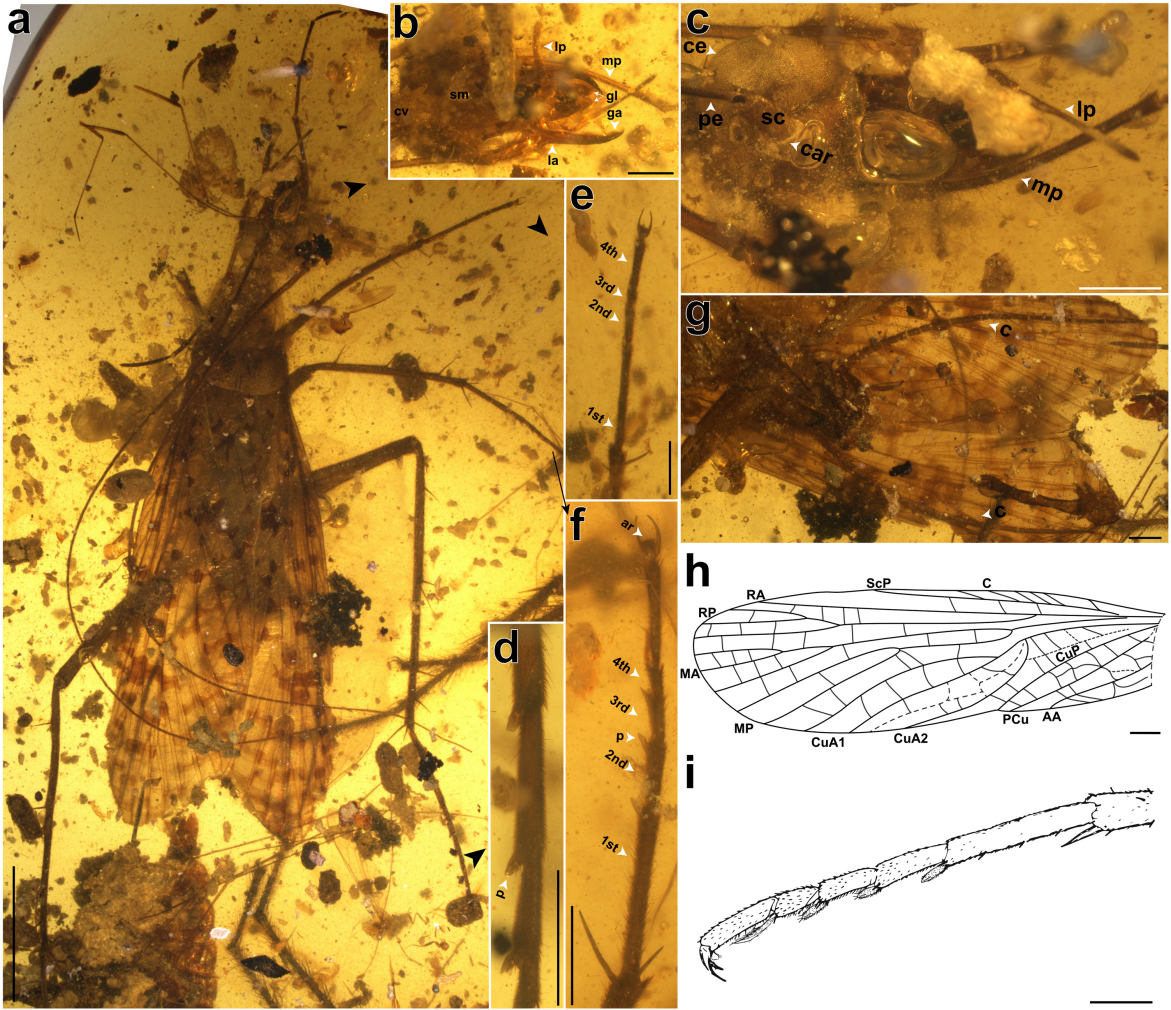
*Zygogrylloblatta longipalpa* sp. nov., male, holotype, NIGP206615. Photographs of (a) dorsal view; (b) dorsal view of head; (c) ventral view of head; (d) pulvilli on metatarsus, arrow indicates a puvillus; (e) protibia and protarsus; (f) mesotarsus; (g) posterior abdomen and cerci. (h) Line drawing of forewing. (i) Line drawing of hindleg. Abbreviations: C, costal vein; ScP, posterior subcosta; RA, anterior branch of radius; RP, posterior radius; MA, anterior branches of media; MP, posterior branches of media; CuA, cubitus anterior; CuA1, first branch of CuA; CuA2, second branch of CuA; CuP, cubitus posterior; PCu, post cubitus; AA, anal anterior. 1st, 2nd, 3rd, 4th, tarsomeres I–iV; ar, arolium; car, circumantennal ridge; c, cerci; ce, compound eye; cv, cervix; ga, galea; la, lacinia; lp, labial palpus; mp, maxillary palpus; p, pulvillus; pe, pedicellus; gl, glossa; sc, scape; sm, submentum. Scale bars, 1 mm.

**Figure 2.**
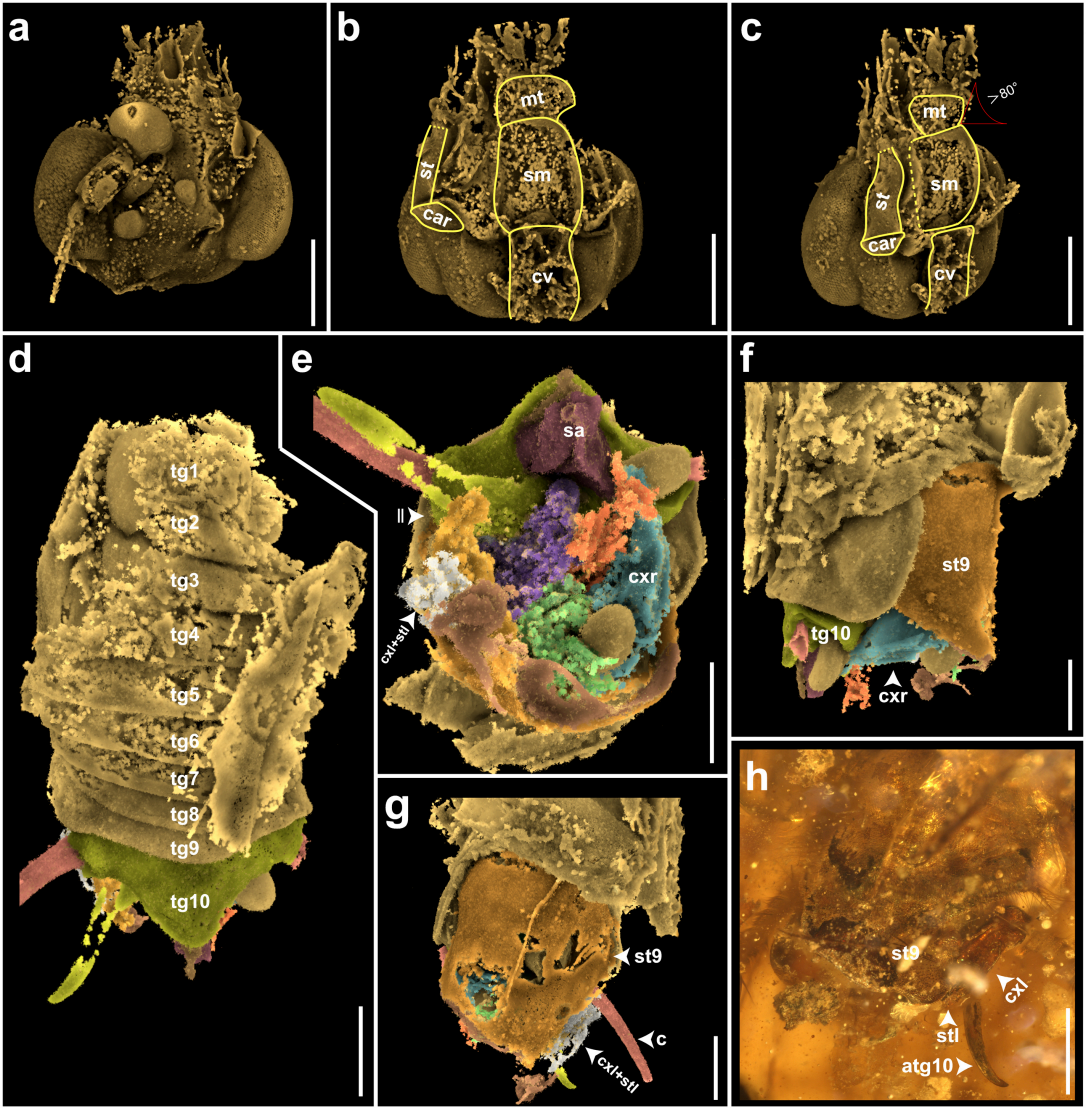
Three-dimensional reconstructions from micro-computed tomography data showing head and male terminalia structures of *Zygogrylloblatta longipalpa* sp. nov., where each structure is identified with a different colour. (a–c) Dorsal, ventral and semi-lateral views of head. (d) Abdomen (ventral view). (e–h) Male terminalia from different views. Abbreviations: atg10, arm of 10th tergite; c, cercus; car, cardo; cv, cervix; cxr, cxl, right and left coxites; ll, left lobe phallus; tg1−10, 1st−10th tergite; mt, mentum; sa, supra-anal plate; st, stipes; stl, apical styli; sm, submentum; st9, 9th sternite. Scale bars, 1 mm.

*Material*. Holotype. ♂, NIGP206615 (a well preserved winged male adult), deposited in Nanjing Institute of Geology and Palaeontology, Chinese Academy of Sciences (NIGPAS).

*Type locality and horizon*. Hukawng Valley, Kachin State, northern Myanmar; upper Albian–lower Cenomanian (*ca* 98.79 ± 0.62 Ma).

*Etymology*. The specific epithet refers to the extremely elongate maxillary palpus.

*Diagnosis*. As for the genus (*vide supra*).

*Description*. See electronic supplementary material, data S5 for the complete description.

*ZooBank LSID*: urn:lsid:zoobank.org:act:3560D03C−93BA−47B7−8B82−528489616FA7

**Remarks.**

Until recently, the Kachin amber ‘Grylloblattodea’ were known from only two fossil species. Zhang *et al*. [36] described a ‘grylloblattodean’ nymph as *Sylvalitoralis cheni* Zhang, Bai, & Yang, 2016. Although young nymphs are hardly comparable to adults, it differs from the new fossil in the presence of only nine cercomeres and the spines of the metafemur that are not grouped. Storozhenko & Gröhn [8] described an adult Kachin amber ‘grylloblattodean’ as *Aristovia daniili*, placing it in its own family, Aristoviidae. These authors characterized this family as follows: ‘most similar to the Early Permian–Middle Triassic Megakhosaridae and the Middle Permian–Middle Jurassic Blattogryllidae in the large head with relatively large compound eyes and three small ocelli, filiform multi-segmented antennae, orthopteroid mouthparts, 5-segmented tarsi, and wing venation, but differs from both by lacking the paranotalia (lateral expansions of the pronotum) and by Sc ending on RA near the apex in both fore- and hind wings’ [8, p. 166]. The new fossil shares with *Aristovia* the rather large head with large compound eyes and the very narrow paranotalia, but *Aristovia* differs notably from the new fossil in the quite shorter maxillary palpi, about as long as the mandible, forewing with a longer ScP, CuA fused basally with M, and the distinctly narrower area between CuA and CuP. The new fossil also differs from the Blattogryllidae and Plesioblattogryllidae Huang, Nel & Petrulevičius, 2008 in having CuA and M independent [15,16,38,39]. Thus, the new fossil cannot be attributed to the Blattogryllidae or the Aristoviidae. Indeed, the distinctions between these small extinct families are insufficient for such taxonomic splitting and all are here combined as the single family Blattogryllidae.

Only *Juraperla daohugouensis* Huang & Nel, 2007 [37] (in Necrophasmatidae Martynоv, 1925 after [40]), is known to have three (or four?) tarsomeres in at least one leg, while fossil ‘Grylloblattodea’ for which the tarsomeres are preserved have five. Unfortunately, the number of tarsomeres in other Necrophasmatidae is unknown [40]. This reduction of the number of tarsomeres could be a putative synapomorphy of the new fossil with the Triassic and Jurassic Necrophasmatidae, but the convergent or independent reduction of number of tarsomeres is a rather common phenomenon across insect clades [41–43] and therefore this character alone is insufficient to consider the new fossil in a clade with Necrophasmatidae.

### (b) Phylogenetic reconstruction

The core of our phylogenetic reconstruction was to test the apomorphies for extant and extinct Grylloblattodea within the broader phylogenetic framework of Polyneoptera. To achieve this, we carefully selected fossil grylloblattodeans with well observable body structures in addition to accurate wing characteristics, so that we could establish accurate morphological homologies between winged and wingless species of Grylloblattodea.

**Figure 3.**
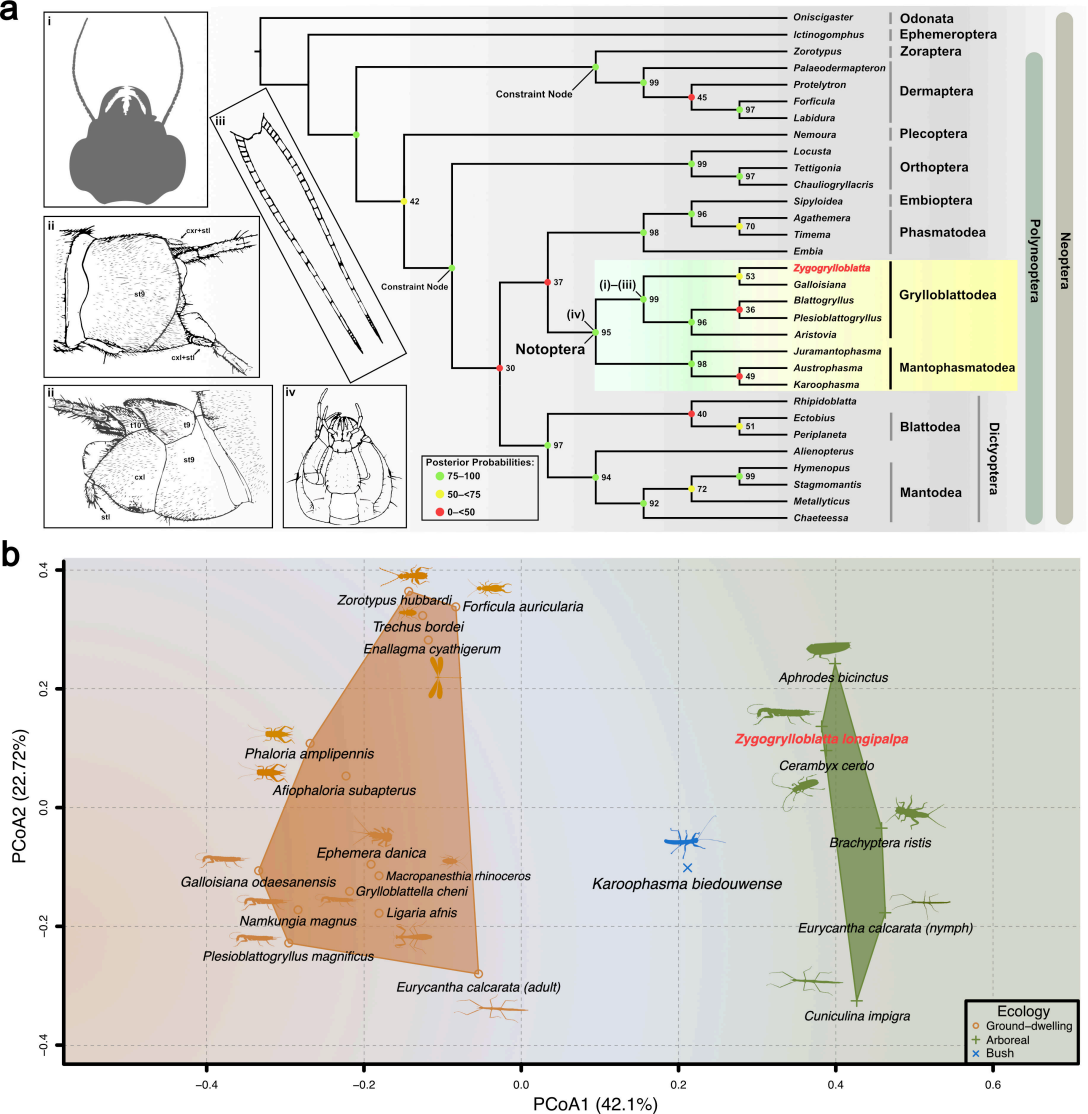
(a) Consensus tree obtained from Bayesian analysis with Polyneoptera node topological constraints according to the phylogenetic results of Wipfler *et al.* [6]. Posterior probability values shown next to node, new genus shown in bold red. (i–iv) Illustrations: (i) silhouette of head; (ii) male terminalia from different views; (iii) cerci; (iv) head, ventral view. For complete phylogenetic resampling results and apomorphies map, see electronic supplementary material, data S3, figures S1–S4. (b) Ordination plot of principal coordinates analysis (PCoA) of insect leg characters, new species shown in bold red. Abbreviations: cxr, cxl, right and left coxites; st9, 9th sternite; stl, apical styli; t9, t10, tergite. For list of specimens and discrete morphological characters see electronic supplementary material, data S4. Insect silhouettes from http://phylopic.org/licences at https://creativecommons.org/publicdomain/zero/1.0/.

In our parsimony and Bayesian phylogenetic analyses conducted at the scale of Polyneoptera, we recovered the same topologies with a monophyletic Notoptera ( = Xenonomia, a junior name and synonym), thereby minimizing an unnecessary proliferation of small, minor orders for much of the fossil diversity (figure 3a). The sister-group relationship of Grylloblattodea+Mantophasmatodea (posterior probability, PP = 0.96) was supported by three synapomorphies (27, 31, 35), of which a distinct angle (more than 60°) between the submentum and mentum, and a submentum curved in lateral view, which are also observed in the new fossil. Regardless of whether the topological relationships of Polyneoptera were constrained, *Zygogrylloblatta* was nested within Grylloblattodea (PP = 0.99) along with extant genera and several Mesozoic fossil ‘Grylloblattodea’ in a single clade (PP = 0.54), supported by one synapomorphy in the male terminalia (61) and seven (2, 21, 22, 23, 24, 53, 62; see electronic supplementary material, data S3; figure S4) homoplasious characters mainly concerning mouthparts. In all results, *Zygogrylloblatta* was recovered as sister to extant Grylloblattidae. Unsurprisingly, internal nodes of Grylloblattodea did not receive strong support as our matrix was constructed to explore the relationship between winged and wingless ice-crawlers, and not to resolve relationships within the crown group Grylloblattodea.

### (c) Palaeoecology of *Zygogrylloblatta*

We conducted a multidimensional scaling using principal coordinates analysis (PCoA) to examine leg features among 20 different species. We selected representative species to reflect the differences in ecological niches, and used descriptive discrete data to quantify leg features. This approach helps to avoid confusion caused by small morphological variations and structural distortions in the images. The PCoA results showed that *Zygogrylloblatta*’s tarsal morphology is closer to that of arboreal species and significantly different from the ground-dwelling extant ice-crawlers (figure 3b). Morphological differences between arboreal and ground-dwelling groups were further supported by a permutational multivariate analysis of variance (PERMANOVA; *F* = 2.516, *p* = 0.0067), suggesting that this morphological distinction reflects genuine ecological divergence rather than random variation. Meanwhile, the Jurassic species *Plesioblattogryllus* Huang & Nel, 2008 [37] was found to be morphologically closer to extant ice-crawlers in terms of its tarsal and pretarsal claw anatomy, suggesting that *Plesioblattogryllus*, like extant ice-crawlers, was unable to move on leaves and plant stems. However, this result may be confounded by fossil preservation and palaeoenvironmental differences, necessitating further evidence for corroboration. The position of the bush-crawler (*Karoophasma biedouwense* Klass, 2003 [4]) was found intermediate between the two ecological niches, which is congruent with its behaviour and ecology.

## 3. Discussion

### (a) Systematics of Notoptera: relationships and apomorphies

**Figure 4.**
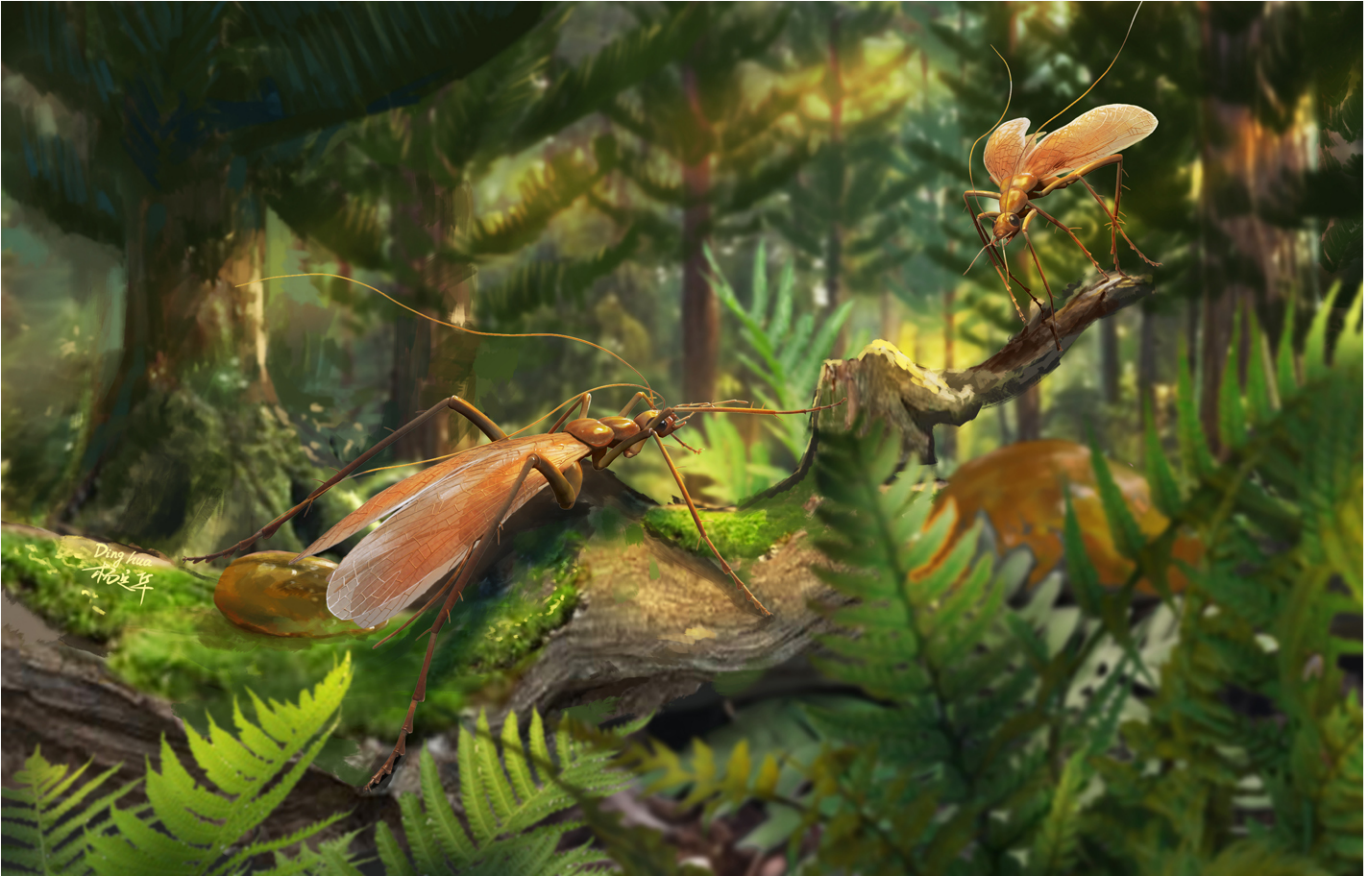
Palaeoecological reconstruction of *Zygogrylloblatta longipalpa* sp. nov. in a Mesozoic forest. Created by Mr Dinghua Yang.

Hitherto, relationships between fossil and extant ice-crawlers have remained uncertain and lack shared synapomorphies owing to the frequent absence of preserved morphological details in compression fossils. The new material and, emphatically, the use of three-dimensional rendering to explore the morphology reveal key morphological details for this fossil Grylloblattodea. More generally, the new fossil differs from all fossil ‘grylloblattodean’ families by the following combination of characters: maxillary palpus extremely long (a putative autapomorphy); protarsomere tetramerous; no ‘false costa’; RP with three short, apical branches; M and CuA separated; a broad area between CuA and CuP; basal stem of CuA nearly straight; CuA1 with only two branches; CuA2 forming a strong basal curve (a character present in some Geinitziidae, e.g. *Shurabia minuta*); ScP short, only reaching two-thirds of wing length; rather few simple crossveins between main veins. We assembled a morphological matrix and mapped apomorphies on the strict consensus tree from parsimony analysis (electronic supplementary material, data S3; figures S2 and S4). The new fossil has a forewing venation globally similar to those of the other fossil winged ‘Grylloblattodea’, especially those with a division of CuA into a branched CuA1 and a simple CuA2, suggesting that at least some other fossil representatives of ‘Grylloblattodea’ also belong to a clade with the new fossil and Grylloblattidae. Additionally, the body features of the new fossil—such as the head, pentamerous tarsi on mid and hind legs, male genitalia and cerci—align with extant Grylloblattidae. Our parsimony analysis further defines the status of these apomorphies linking winged and wingless ice-crawlers (figure 3a(i–iii); electronic supplementary material, data S3; figures 2 and 4, electronic supplementary material, figures S2 and S4), challenging previously suggested synapomorphies:

(i) *Zygogrylloblatta* has a sickle-shaped lacinia with a clearly convex mesal margin lacking setae, and a sickle-shaped galea with a mesial brush of setae (figure 3a(i)), which is the same as in extant *Grylloblatta campodeiformis*. These are not unique to Grylloblattidae among extant Polyneoptera, as sickle-shaped laciniae and galeae are not uncommon among earwigs [44,45], such as *Forficula auriculata*. The galea and lacinia can significantly vary in shape and ornamentation among extant Grylloblattidae, *viz*. in *Galloisiana* versus *Grylloblatta* [46], figure 2c,d. Thus, these two characters are not stable among extant Grylloblattidae and they cannot be considered as strictly present in this group and therefore may not be synapomorphies of Grylloblattodea;
(ii) The terminalia of the male of *Zygogrylloblatta* align closely with those of extant Grylloblattidae (figure 3a(ii)), as described by Walker [47, pl. 8; 48, pl. 4]. In Grylloblattodea, coxae IX (or coxopods IX) are distinct from sternum IX, which is likely a derived state given the deeply nested evolutionary position of the order [49]. In contrast, in other orders such as Dermaptera, some Orthoptera and Dictyoptera, while proximally articulated styli of segment IX are present, coxae IX are not differentiated from sternum IX [50, p. 576, fig. 17]. The presence of asymmetrical external male terminalia of Grylloblattodea, characterized by well developed ninth-segmental coxae bearing distinct styli, is unique within the Polyneoptera [50, pp. 577−578]. It is important to note that the presence of ninth-segmental coxae alone is not a distinguishing character, as this is likely a symplesiomorphy common to Insecta. However, the asymmetry of these structures is considered apomorphic [38]. Therefore, it is the combination of separated and asymmetric coxae that distinctly characterizes Grylloblattidae. Our study shows that the ninth-segmental coxae (cx l and cx r) of *Grylloblatta campodeiformis* precisely match the structures observed in the new fossil [48, pl. 4, figs 12–14]. Consequently, we propose that these coxae with apical styli represent the first synapomorphy linking a winged Mesozoic ‘Grylloblattodea’ with extant Grylloblattidae;
(iii) Other potential synapomorphies include the reduction of the arolium, a long ovipositor and the multiarticulated cerci [25]. We can clearly observe the presence of arolia in several other fossil Grylloblattodea, including *Zygogrylloblatta* (figure 3a(iii)), and this character is probably related to the mode of life of these insects. The long ovipositor and multimerous cerci are present in extant Grylloblattidae, but using these characters as apomorphies does not adequately take into account Palaeozoic or Mesozoic Dictyoptera [51], also with elongate ovipositors and long, multimerous cerci, such as *Rhipidoblatta* [52] (used in the morphological analysis), which thus renders the polarization of this character uncertain. Accordingly, it remains unclear whether such homoplasic traits are locally apomorphic within Grylloblattodea or have evolved multiple times independently across Polyneoptera;
(iv) The three-dimensional rendering of the head of *Zygogrylloblatta* shows that the mentum makes an angle of approximately 80° with the submentum and the labial palpi are oriented posteriorly (figures 2c and 3a(iv)). These two characters are synapomorphies of the Notoptera (Grylloblattodea + Mantophasmatodea) [6,53,54];
(v) A potential synapomorphy of Notoptera is the complete loss of wings, but that state is not supported by the fossil record, with a wingless Mantophasmatodea from the Jurassic but winged Grylloblattodea fossils [7,11,55]. It is thus reasonable to assume that the loss of wings is not a synapomorphy of Notoptera, but rather an independently derived character state in both lineages.

By this, we demonstrate that the presence of coxae with apical styli is the first synapomorphy defining relationships between extant and fossil Grylloblattodea, and that the characters ‘mentum making an angle of *ca* 80° with the submentum and labial palpi oriented posteriorly’ present in the new fossil supports the monophyly of Notoptera.

### (b) Multiple ecological transitions within Grylloblattodea

*Zygogrylloblatta* inhabited warm, humid tropical environments [56], a stark contrast to the high-latitude, high-elevation habitats of extant Grylloblattidae in the Northern Hemisphere [7,30]. The divergence time of the extant Grylloblattidae is unknown, dated to 30−115 Ma [57], but it may overlap with the age of the Burmese amber, which could suggest that the ice-crawlers preserved in Burmese amber are from the early period of divergence of the extant linages. Ecological shifts in extant ice-crawlers have often been correlated with significant environmental changes in the high latitudes of the Northern Hemisphere [11,30,35,58,59]—the breakup of the Japanese islands from the Asian continent [57], the advance and retreat of North American icebergs [27], the barrier of the Altai Mountains, and the intermittent Beringian land bridge [35,58]—all occurring after the late Palaeogene (*ca* 33.9 Ma). Notably, all fossil Grylloblattodea have thus far been associated with warm and sometimes humid environments [16,49,60], suggesting that the crown-group ice-crawlers may have adapted to colder habitats following the Grand Coupure global cooling event about 34 million years ago [61]. The >50 million-year gap (mid-Cretaceous to mid-Palaeogene) suggests the existence of a ghost lineage bridging tropical ancestors and cryophilic descendants—a hypothesis now supported by *Zygogrylloblatta*’s unique combination of ancestral and derived traits, which may illuminate our understanding of early ecological shifts in this group.

A remarkable feature of *Zygogrylloblatta* is its exceptionally elongated maxillary palpi, which surpass those of all known extant and extinct Grylloblattodea. In insects, maxillary palpi hold chemosensory receptors. Their elongated shape in *Zygogryloblatta* could be related to their feeding habits or species recognition abilities [62–64]. *Zygogrylloblatta* also possesses a strengthened distal spur on the tibia, a flattened tarsus with euplantulae and a pretarsus with stout paired claws and an arolium that does not curve ventrally. While these morphological features are theoretically compatible with movement on diverse substrates (e.g. plant surfaces, rough bark, stones or irregular terrains), they strongly suggest an arboreal ecology for *Zygogrylloblatta*, with euplantulae and adhesive arolium convergent with arboreal polyneopterans (e.g. some arboreal leafhoppers [65] and stick insects [66,67]) specialized for plant substrate navigation [43,68,69]. Locomotor attachment devices on legs, such as arolia and euplantulae, have played a critical role in the evolution of highly successful insects, providing essential frictional forces for movement across various surfaces and enabling efficient walking on plants through a rapid, reversible attachment/detachment system [65–68,70–72]. The variations in these structures among different insects reflect differences in their behaviour and ecological habits [65–67,73]. Extant Grylloblattidae have variable or absent euplantulae [10,46] and are all ground-dwelling. Among polyneopterans, only Zoraptera lack tarsal euplantulae [6], suggesting that a trend of euplantulae reduction across polyneopteran groups is a convergent adaptation to habitats. In Grylloblattidae, reduced tarsal pads likely facilitate life on rocky ground, where minimal attachment is advantageous. In contrast, Mantophasmatodea, which retains well developed tarsal attachment pads, likely benefits from improved grip that aids in climbing through bushes and low vegetation [54]. This difference in tarsal morphology highlights functional adaptations to their respective habitats.

Although *Zygogrylloblatta* shared the arboreal niche with certain Orthoptera [74], Phasmatodea [66] and Hemiptera [65], these orders survived multiple mass extinctions and continue to thrive in modern ecosystems [75–77]. In contrast, winged ice-crawlers disappeared following the mid-Cretaceous, although the exact timing of their extinction remains unknown [55]. Extant Grylloblattidae have structured geographical patterns and a generalized body structure, favouring cool and moist microhabitats in mountainous regions, where they secure nutritional resources while avoiding intense competition [3,32]. A plausible hypothesis to explain this distribution is that mid-Cretaceous Grylloblattodea adapted to environmental fluctuations by migrating along thermal gradients. This migration would have contributed to their colonization and diversification in regions such as the Altai Mountains, Siberia and the northwest Cordillera Mountains, USA and Canada. The biogeographical distribution of extant Grylloblattidae suggests that wing loss and adaptation to narrow, extreme habitats occurred later, likely following global cooling events at the end of the Palaeogene [61]. These same climatic pressures may have independently driven wing loss in both extant Grylloblattidae and Mantophasmatodea [7,11,34].

In summary, based on morphological analysis, *Zygogrylloblatta* represents a lineage of winged, arboreal ‘ice’-crawlers—a somewhat misleading term, as it lived in warm, humid, tropical environment. This species was supposedly well adapted for agile movements and predation. Its specialized legs enabled efficient movements through Mesozoic terrestrial ecosystems, allowing it to thrive amidst considerable competition (figure 4). Although the factors driving ancient extinction patterns within Notoptera remain poorly understood owing to limited fossil evidence, *Zygogrylloblatta* provides a crucial insight into the evolutionary history of ice-crawlers. Its adaptations reflect the ecological transitions and selective pressures that shaped this group, highlighting the role of unique morphological and functional traits in their evolutionary trajectory.

## 4. Methods

### (a) Origin and imaging of the amber piece

The ice-crawler is preserved at the centre of a large, irregularly shaped piece of Kachin amber that is 57 mm long, 38 mm wide and 8 mm high, 11.5 g weight. Shi *et al*. [78] used U–Pb isotope dating to determine the age of the Kachin amber to be 98.79 ± 0.62 Ma [78]. Two additional sets of dating results from ammonites and ostracods captured in Kachin amber also support this conclusion [56,79]. This ice-crawler has morphological details of the body, wings and appendages preserved (the ice-crawler’s abdomen is slightly compressed without affecting its integrity), but some details covered by mineral grains, gas bubbles, plant and insect debris and its own legs (figure 1a). In addition to the ice-crawler, the piece also contains several hymenopteran wings, plant debris, mud particles and a spider (figure 1a). The amber is deposited at NIGPAS. Photographs were taken using a Zeiss Stereo Discovery V16 Microscope System in NIGPAS, and measurements were taken using Zen software. Photomicrographic composites of 10−150 individual focal planes were digitally stacked using the software Helicon Focus 6.7.1 for a better illustration of three-dimensional structures. Figures were made in Adobe Illustrator CC and Adobe Photoshop CS5 (Adobe Systems, San José, USA). The classification adopted here follows that of Grimaldi & Engel [2] and Storozhenko [16,23], while the morphological terminology of head and postabdominal elements follows Walker [48] and Wipfler *et al.* [53], and the wing venation nomenclature generally follows Storozhenko [16] and Cui *et al.* [24], modified to integrate the existence of the vein PCu in the Neoptera [80].

### (b) Micro-computed tomography scanning

The specimen was scanned at the micro-computed tomography (micro-CT) laboratory of Yunnan Key Laboratory for Palaeobiology with an Xradia 520 Verse X-ray microscope (3D-XRM; Carl Zeiss X-ray Microscopy, Pleasanton, USA. We performed a total of four scans of this specimen, including a low-resolution whole-body scan and high-resolution scans of three different locations (head, terminal abdomen and tarsus). Volume rendering results indicate that the specimen retains sufficient identification information of its external morphology at the head and male terminalia. The CT-scan parameters of this specimen are provided in electronic supplementary material, data S1. We identified various structures of the male genitalia with different colours in volume rendering. Three-dimensional reconstruction, volume rendering, rendering images and video generation of the specimen were completed using the open-source software Drishti 3.2 [81].

### (c) Phylogenetic reconstruction

#### (i) Taxonomic sampling

Phylogenetic estimation of relationships among Polyneoptera including Grylloblattodea was based on the sampling of Cui *et al.* [25]. However, we modified the sampling by deleting three outgroups, phylogenetically distant from Polyneoptera, retaining only one Palaeoptera and one Holometabola. The removal of these outgroups does not affect the polarization of the characters or testing of monophyly for the ingroup. We improved the taxonomic sampling by adding the new fossil, *Zygogrylloblatta longipalpa* gen. et sp. nov., and *Plesioblattogryllus magnificus*. In addition, we added fossils that possess interesting features for the phylogeny of Polyneoptera, such as *Blattogryllus karatavicus* Rasnitsyn, 1976 [15] (Grylloblattodea) with male genitalia partly preserved, and *Rhipidoblatta fusca* Handlirsch, 1906 [82] (Dictyoptera), which preserves a long female ovipositor. Lastly, we added two fossils belonging to extant polyneopteran orders: *Juramantophasma sinica* Huang, Nel, Zompro & Waller, 2008 [83] (Notoptera: Mantophasmatodea) and *Palaeodermapteron dicranum* Zhao, 2011 [84] (Dermaptera).

#### (ii) Morphological dataset

The morphological matrix encompassed 81 characters. To the 71-character matrix of Cui *et al.* [25], we added 10 characters extracted from the existing literature (electronic supplementary material, data S2) that we considered informative to assess the phylogeny of winged ice-crawlers. Seventy-five characters are binary and six of them are multi-stated characters; all characters are unordered, non-polarized and of equal weight. Characters coded as inapplicable were treated as missing data and coded ‘–’, while missing data were coded ‘?’. The morphological matrix is available in electronic supplementary material, data S3.

#### (iii) Parsimony analysis

The parsimony analysis was performed with TNT [85] (version 1.6) software. The research of the most parsimonious trees was made with New Technology Analysis (Sectorial Search, Ratchet, Drift, TreeFusing), with 100 Random Addition Sequence (RAS) and Tree Bisection–Reconnection parameters (TBR); two outgroups were used to polarize the transformations and root the tree; all the characters were treated unordered and the transformations equally; the two outgroups were treated as paraphyletic with respect to the ingroup. All characters were weighted using implied character weighting options. Implied weighting (IW) analysis uses the TNT script setk.run (author: Salvador Arias) to calculate the optimal concavity constant value (*K*-value). This value plays a role in defining the weight of homoplasic characters based on the degree of similarity in implicit weighting [86], which can optimize the results of phylogenetic analysis [85]. In the initial agnostic search, the branching nodes of certain family-level taxa were questionable. Therefore, we enforced the topological relationships of these taxa in subsequent optimized searches based on the phylogeny of Wipfler *et al.* [6], which resulted in consistent phylogenetic relationships of the nodes within Grylloblattodea across the two searches. To evaluate the support of our phylogenetic hypothesis, we conducted a bootstrap analysis [87] by using 10 000 technical pseudo-replications, and reported each value. We reconstructed the strict consensus that collapses all the branches that are found in fewer than 100% of the trees. The final results were edited and enhanced using Winclada [88] (version 1.7) software and Adobe Illustrator CC software.

#### (iv) Bayesian phylogenetic analysis

To verify and strengthen the results of the parsimony analysis, we repeated the phylogenetic analysis using Bayesian phylogenetic analysis, again including unconstrained and constrained nodes within Polyneoptera, and the results were consistent with those of the parsimony analysis. The Bayesian phylogenetic inferences were performed with MrBayes (version 3.2.7.a) software [89–91]. We carried out our analysis with a Markov one-parameter (Mkv) model [92], with a gamma rate variation across characters. Each search comprised two runs and four Markov chain Monte Carlo (MCMC) and was launched for 20 million generations. The MCMCs were sampled every 5000 generations, and a burn-in fraction of 0.25 was used. Convergence diagnostics were checked for each analysis, with the average standard deviation of split frequencies (ASDSF) <0.01, potential scale reduction factor (PRSF) close to 1.0 in outputs, and an effective sample size (ESS) >200 in Tracer v.1.7.1 software [93]. Posterior probabilities (PP) are used to discuss the relationships’ support.

### (d) Comparative analysis of locomotor attachment structure: principal coordinate analysis and permutational multivariate analysis of variance

We selected 16 leg characters from 20 species for principal coordinate analysis (PCoA) (figure 3b and electronic supplementary material, data S3). The selection included two fossils and 18 extant species. The fossil species included the new fossil, and *Plesioblattogryllus* (*Plesioblattogryllus magnificus*; Jurassic; Daohugou locality, China), which possesses characters typically associated with extant ice-crawlers. Extant species were selected separately according to their ecological niches: ground-dwelling species included three extant adult ice-crawlers and a representative species of the orders Blattodea, Ephemeroptera, Dermaptera, Coleoptera: Carabidae, and Zoraptera; arboreal species included a leafhopper, a bush cricket and two phasmatodeans, two species of grass crickets, and a representative species of the orders Plecoptera, Coleoptera and Odonata; as well as a ground- and low-bush-living bush-crawler *Karoophasma biedouwense* Klass, 2003 [4]. Overall, our sampling included most of the representative orders of Polyneoptera as well as a few species in Neoptera that have well defined ecological niches and records. Notably, among arboreal species, the tarsal and pretarsal attachment pads of *Eurycantha calcarata* Lucas, 1869 [94] (Phasmatodea) change strongly during postembryonic development, biased towards leaf-dwelling in the juvenile stage and towards ground-dwelling as it changes in size to the adult stage. The selection of this species (coded as two terminals, as nymph and adult) contributes to the reliability of the results of our assay.

Morphological characters and descriptions were obtained from the literature (see electronic supplementary material, data S4). The PCoA of the characters of legs were conducted in PAST [95] (Palaeontological Statistics) 3.15 and R [96] using the packages ape [97] for PCoA analysis and vegan [98] for the distance matrix calculation. We calculated the similarity matrix using the Jaccard distance [99] for discrete traits. We performed the PCoA analysis and kept the two main axes (figure 3b).

To assess the morphological differences between arboreal and ground-dwelling insects, we employed a single-variable permutational multivariate analysis of variance (PERMANOVA). The analysis was conducted using the PAST [95] software. We computed a distance matrix using the Jaccard dissimilarity measure, suitable for discrete data. The PERMANOVA was performed with 9999 permutations to ensure robustness of the results. A *p*-value of less than 0.05 indicates a significant difference between the different groups.

## Data Availability

All of the processed data associated with this study are available on Figshare [100]. Supplementary material is available online [101].

## References

[1] Walker EM. 1932 Classification of insects, a key to the known families of insects and other terrestrial arthropods. Ann. Entomol. Soc. Am. **25**, 262–263. (10.1093/aesa/25.1.262c)

[2] Grimaldi DA, Engel MS. 2005 Evolution of the insects. Cambridge, UK: Cambridge University Press.

[3] Wipfler B, Bai M, Schoville S, Dallai R, Uchifune T, Machida R, Cui Y, Beutel RG. 2014 Ice crawlers (Grylloblattodea) – the history of the investigation of a highly unusual group of insects. J. Insect Biodivers. **2**, 1–25. (10.12976/jib/2014.2.2)

[4] Klass KD, Zompro O, Kristensen NP, Adis J. 2002 Mantophasmatodea: a new insect order with extant members in the Afrotropics. Science **296**, 1456–1459. (10.1126/science.1069397) 11964441

[5] Misof B *et al*. 2014 Phylogenomics resolves the timing and pattern of insect evolution. Science **346**, 763–767. (10.1126/science.1257570) 25378627

[6] Wipfler B *et al*. 2019 Evolutionary history of Polyneoptera and its implications for our understanding of early winged insects. Proc. Natl Acad. Sci. USA **116**, 3024–3029. (10.1073/pnas.1817794116) 30642969 PMC6386694

[7] Schoville SD. 2014 Current status of the systematics and evolutionary biology of Grylloblattidae (Grylloblattodea). Syst. Entomol. **39**, 197–204. (10.1111/syen.12052)

[8] Storozhenko SYU, Gröhn C. 2023 A new family of grylloblattids (Insecta: Grylloblattida) from mid-Cretaceous Burmese amber. Palaeoentomology **6**, 165–173. (10.11646/palaeoentomology.6.2.8)

[9] Zhou L, Wang T, Liu YI, Qi Y, Chen QI, Ren B. 2025 New data on Galloisiana sinensis Wang, 1987 (Grylloblattodea: Grylloblattidae) with description of the hitherto unknown female. Zootaxa **5584**, 270–280. (10.11646/zootaxa.5584.2.7) 40174076

[10] Zhou L, Chen Q, Ke H, Wang Z, Peng J, Wu D, Liu Y, Feng J, Ren B. 2023 Descriptions of a new genus and a new species, Grylloprimevala jilina (Grylloblattidae) from China. Ecol. Evol. **13**, e9750. (10.1002/ece3.9750) 36699568 PMC9852939

[11] Schoville SD *et al*. 2021 Comparative transcriptomics of ice‐crawlers demonstrates cold specialization constrains niche evolution in a relict lineage. Evol. Appl. **14**, 360–382. (10.1111/eva.13120) 33664782 PMC7896716

[12] Terry MD, Whiting MF. 2005 Mantophasmatodea and phylogeny of the lower neopterous insects. Cladistics **21**, 240–257. (10.1111/j.1096-0031.2005.00062.x)

[13] Crampton GC. 1915 The thoracic sclerites and systematic position of Grylloblatta campodeiformis Walker, a remarkable annectant orthopteroid insect. Entomol. News **26**, 337–350.

[14] Carpenter FM. 1992 Treatise on invertebrate paleontology, pt R, Arthropoda 4, vols 3 and 4. Superclass Hexapoda. Boulder, Colorado: Geological Society of America. (10.17161/dt.v0i0.5370)

[15] Rasnitsyn A. 1976 [Grylloblattidae - living representatives of order Protoblattodea (Insecta).]. Dokl. Akad. Nauk SSSR **228**, 502–504.

[16] Storozhenko SI. 1998 Sistematika, filogenii︠a︡ i ėvoli︠u︡t︠s︡ii︠a︡ grilloblattidovykh nasekomykh (Insecta: Grylloblattida) [Systematics, classification and distribution of grylloblattid insects (Insecta:Grylloblattida)]. Vladivostok, Russia: Dalʹnauka.

[17] Aristov D. 2015 Classification of the order Eoblattida (Insecta: Blattidea) with description of new taxa. Far East. Entomol. **301**, 1–56.

[18] Aristov DS. 2017 Palaeozoic evolution of the Insecta Gryllones. PhD thesis, Moscow, Federal State Budgetary Institution of Science A.A. BORISYAK PALEONTOLOGICAL INSTITUTE OF THE RUSSIAN ACADEMY OF SCIENCES.

[19] Arillo A, Engel M. 2006 Rock crawlers in Baltic amber (Notoptera: Mantophasmatodea). Am. Mus. Novit. **3539**, 1–10. (10.1206/0003-0082(2006)3539[1:RCIBAN]2.0.CO;2)

[20] Béthoux O, Nel A. 2002 Venation pattern and revision of Orthoptera sensu nov. and sister groups. Phylogeny of Palaeozoic and Mesozoic Orthoptera sensu nov.. Zootaxa **96**, 1–88. (10.11646/zootaxa.96.1.1)

[21] Schubnel T, Roberts D, Roques P, Garrouste R, Desutter-Grandcolas L, Nel A. 2020 Moscovian fossils shed light on the enigmatic polyneopteran families Cacurgidae and Eoblattidae (Insecta: ‘Eoblattida’, Archaeorthoptera). J. Syst. Palaeontol. **18**, 499–511. (10.1080/14772019.2019.1627595)

[22] Beutel RG, Friedrich F, Yang X-K, Ge S-Q. 2013 Insect morphology and phylogeny: a textbook for students of entomology. Berlin, Germany: Walter de Gruyter. (10.1515/9783110264043)

[23] Storozhenko SY. 2002 Order Grylloblattida Walker, 1914. In History of insects (eds AP Rasnitsyn, DLJ Quicke), pp. 278–284. Dordrecht, The Netherlands: Kluwer Academic Publishers. (10.1007/0-306-47577-4)

[24] Cui Y, Storozhenko Sy, Ren D. 2012 New and little-known species of Geinitziidae (Insecta: Grylloblattida) from the Middle Jurassic of China, with notes on taxonomy, habitus and habitat of these insects. Alcheringa **36**, 251–261. (10.1080/03115518.2012.628806)

[25] Cui Y *et al*. 2024 A winged relative of ice‐crawlers in amber bridges the cryptic extant Xenonomia and a rich fossil record. Insect Sci. **31**, 1645–1656. (10.1111/1744-7917.13338) 38454304

[26] Mills HB, Pepper JH. 1937 Observations on Grylloblatta campodeiformis Walker. Ann. Entomol. Soc. Am. **30**, 269–275. (10.1093/aesa/30.2.269)

[27] Schoville SD, Graening GO. 2013 Updated checklist of the ice-crawlers (Insecta: Grylloblattodea: Grylloblattidae) of North America, with notes on their natural history, biogeography and conservation. Zootaxa **3737**, 351–378. (10.11646/zootaxa.3737.4.2) 25112759

[28] Schoville SD, Kim BW. 2011 Phylogenetic relationships and relictualism of rock-crawlers (Grylloblattodea: Grylloblattidae) in cave and mountain habitats of Korea. Ann. Entomol. Soc. Am. **104**, 337–347. (10.1603/an10125)

[29] Kamp JW. 1963 Descriptions of two new species of Grylloblattidae and of the adult of Grylloblatta barberi, with an interpretation of their geographical distribution. Ann. Entomol. Soc. Am. **56**, 53–68. (10.1093/aesa/56.1.53)

[30] Kamp J. 1973 Biosystematics of the Grylloblattodea. PhD Thesis, University of British Columbia. (10.14288/1.0101053)

[31] Schoville SD, Roderick GK. 2010 Evolutionary diversification of cryophilic Grylloblatta species (Grylloblattodea: Grylloblattidae) in alpine habitats of California. BMC Evol. Biol. **10**, 163. (10.1186/1471-2148-10-163) 20525203 PMC2898686

[32] Schoville SD, Slatyer RA, Bergdahl JC, Valdez GA. 2015 Conserved and narrow temperature limits in alpine insects: thermal tolerance and supercooling points of the ice-crawlers, Grylloblatta (Insecta: Grylloblattodea: Grylloblattidae). J. Insect Physiol. **78**, 55–61. (10.1016/j.jinsphys.2015.04.014) 25956197

[33] Angilletta Jr MJ, Wilson RS, Navas CA, James RS. 2003 Tradeoffs and the evolution of thermal reaction norms. Trends Ecol. Evol. **18**, 234–240. (10.1016/s0169-5347(03)00087-9)

[34] Schoville SD, Bougie TC, Dudko RY, Medeiros MJ. 2019 Has past climate change affected cold‐specialized species differentially through space and time? Syst. Entomol. **44**, 571–587. (10.1111/syen.12341)

[35] Vrsansky P, Storozhenko S, Labandeira C, Ihringova P. 2001 Galloisiana olgae sp. nov. (Grylloblattodea: Grylloblattidae) and the paleobiology of a relict order of insects. Ann. Entomol. Soc. Am. **94**, 179–184. (10.1603/0013-8746(2001)094[0179:GOSNGG]2.0.CO;2)

[36] Zhang W, Guo M, Yang X, Bai M. 2016 A new species of ice crawlers from Burmese amber (Insecta: Grylloblattodea). Zool. Syst. **41**, 327–331. (10.11865/zs.201637)

[37] Huang DY, Nel A, Petrulevičius JF. 2008 New evolutionary evidence of Grylloblattida from the Middle Jurassic of Inner Mongolia, north-east China (Insecta, Polyneoptera): new Mesozoic grylloblattid family. Zool. J. Linn. Soc. **152**, 17–24. (10.1111/j.1096-3642.2007.00351.x)

[38] Cui Y. 2012 New data on the Blattogryllidae-Plesioblattogryllidae-Grylloblattidae complex (Insecta: Grylloblattida: Blattogryllopterida tax.n.). Arthropod Syst. Phylogeny **70**, 167–180. (10.3897/asp.70.e31760)

[39] Cui Y, Béthoux O, Yang N, Ren D. 2023 New data and systematic considerations on the Blattogryllopterida (Insecta: Grylloblattida) from the Jurassic Daohugou locality in China. Palaeoentomology **6**, 124–132. (10.11646/palaeoentomology.6.2.4)

[40] Aristov DS. 2018 Revision of the family Necrophasmatidae (Insecta: Cnemidolestida). Far East. Entomol. **359**, 7–11. (10.25221/fee.359.2)

[41] Haas F, Gorb S. 2004 Evolution of locomotory attachment pads in the Dermaptera (Insecta). Arthropod Struct. Dev. **33**, 45–66. (10.1016/j.asd.2003.11.003) 18089022

[42] Moreira GRP, Silva DS, Gonçalves GL. 2017 Comparative morphology of the prothoracic leg in heliconian butterflies: tracing size allometry, podite fusions and losses in ontogeny and phylogeny. Arthropod Struct. Dev. **46**, 462–471. (10.1016/j.asd.2017.03.008) 28373032

[43] Beutel R, Gorb S. 2006 A revised interpretation of attachment structures in Hexapoda with special emphasis on Mantophasmatodea. Arthropod Syst. Phylogeny **64**, 3–25. (10.3897/asp.64.e31640)

[44] Walker EM. 1933 On the anatomy of Grylloblatta campodeiformis Walker. 2. Comparisons of head with those of other orthopteroid insects. Ann. Entomol. Soc. Am. **26**, 309–344. (10.1093/aesa/26.2.309)

[45] Walker EM. 1931 On the anatomy of Grylloblatta campodeiformis Walker. 1. Exoskeleton and musculature of the head. Ann. Entomol. Soc. Am. **24**, 519–536. (10.1093/aesa/24.3.519)

[46] Kim BW, Lee W. 2007 A new species of the genus Galloisiana (Grylloblattodea, Grylloblattidae) from Korea. Zool. Sci. **24**, 733–745. (10.2108/zsj.24.733) 17824781

[47] Walker EM. 1919 The terminal abdominal structures of orthopteroid insects: a phylogenetic study. Ann. Entomol. Soc. Am. **12**, 267–316. (10.1093/aesa/12.4.267)

[48] Walker EM. 1943 On the anatomy of Grylloblatta campodeiformis Walker 4. Exoskeleton and musculature of the abdomen. Ann. Entomol. Soc. Am. **36**, 681–706. (10.1093/aesa/36.4.681)

[49] Aristov DS. 2009 Review of the stratigraphic distribution of Permian Grylloblattida (Insecta), with descriptions of new taxa. Paleontol. J. **43**, 643–651. (10.1134/s0031030109060070)

[50] Boudinot BE. 2018 A general theory of genital homologies for the Hexapoda (Pancrustacea) derived from skeletomuscular correspondences, with emphasis on the Endopterygota. Arthropod Struct. Dev. **47**, 563–613. (10.1016/j.asd.2018.11.001) 30419291

[51] Sendi H, Vršanský P, Azar D. 2023 Jordanian–Lebanese–Syrian cockroaches s.s. from Lower Cretaceous amber – monograph. Biologia **78**, 1447–1541. (10.1007/s11756-023-01357-y)

[52] Vishniakova VN. 1968 Mesozoic cockroaches with an external ovipositor and features of their reproduction (Blattodea). In Yurskie nasekomye Karatau [Jurassic insects of Karatau] (ed. BB Rohdendorf), pp. 55–86. Moscow, Russia: Akademiya nauk SSSR Otdelenie Obshchej Biologii.

[53] Wipfler B, Machida R, Müller B, Beutel RG. 2011 On the head morphology of Grylloblattodea (Insecta) and the systematic position of the order, with a new nomenclature for the head muscles of Dicondylia. Syst. Entomol. **36**, 241–266. (10.1111/j.1365-3113.2010.00556.x)

[54] Wipfler B, Klug R, Ge S, Bai M, Göbbels J, Yang X, Hörnschemeyer T. 2015 The thorax of Mantophasmatodea, the morphology of flightlessness, and the evolution of the neopteran insects. Cladistics **31**, 50–70. (10.1111/cla.12068) 34758578

[55] Righetti N, Forni G, Luchetti A. 2024 Mitochondrial phylogenomics supports a Carboniferous origin of Xenonomia. Eur. Zool. J. **91**, 1139–1146. (10.1080/24750263.2024.2409908)

[56] Yu T *et al*. 2019 An ammonite trapped in Burmese amber. Proc. Natl Acad. Sci. USA **116**, 11345–11350. (10.1073/pnas.1821292116) 31085633 PMC6561253

[57] Schoville S, Uchifune T, Machida R. 2013 Colliding fragment islands transport independent lineages of endemic rock-crawlers (Grylloblattodea: Grylloblattidae) in the Japanese archipelago. Mol. Phylogenet. Evol. **66**, 915–927. (10.1016/j.ympev.2012.11.022) 23220515

[58] Jarvis K, Whiting M. 2006 Phylogeny and biogeography of ice crawlers (Insecta: Grylloblattodea) based on six molecular loci: designating conservation status for Grylloblattodea species. Mol. Phylogenet. Evol. **41**, 222–237. (10.1016/j.ympev.2006.04.013) 16798019

[59] Kamp JW. 1979 Taxonomy, distribution, and zoogeographic evolution of Grylloblatta in Canada (Insecta: Notoptera). Can. Entomol. **111**, 27–38. (10.4039/ent11127-1)

[60] Aristov DS. 2010 Review of grylloblattid insects (Insecta: Grylloblattida) from the Solikamsk deposits of the Perm Region. Paleontol. J. **44**, 505–514. (10.1134/s0031030110050059)

[61] Foottit R, Adler P. 2009 Insect biodiversity: science and society, 1st edn. Oxford, UK: Blackwell Publishing. (10.1002/9781118945582)

[62] Chapman R, De Boer G (eds). 1995 Regulatory mechanisms in insect feeding. Boston, MA: Springer. (10.1007/978-1-4615-1775-7)

[63] Krenn H (ed.). 2019 Insect mouthparts: form, function, development and performance. Cham, Switzerland: Springer International Publishing. (10.1007/978-3-030-29654-4)

[64] Prakash S, Mendki MJ, Rao KM, Singh K, Singh RN. 1995 Sensilla on the maxillary and labial palps of the cockroach Supella longipalpa fabricius (Dictyoptera: Blattellidae). Int. J. Insect Morphol. Embryol. **24**, 13–34. (10.1016/0020-7322(94)00009-f)

[65] Clemente CJ, Goetzke HH, Bullock JMR, Sutton GP, Burrows M, Federle W. 2017 Jumping without slipping: leafhoppers (Hemiptera: Cicadellidae) possess special tarsal structures for jumping from smooth surfaces. J. R. Soc. Interface **14**, 20170022. (10.1098/rsif.2017.0022) 28468924 PMC5454290

[66] Bußhardt P, Wolf H, Gorb SN. 2012 Adhesive and frictional properties of tarsal attachment pads in two species of stick insects (Phasmatodea) with smooth and nubby euplantulae. Zoology **115**, 135–141. (10.1016/j.zool.2011.11.002) 22578997

[67] Grohmann C, Henze MJ, Nørgaard T, Gorb SN. 2015 Two functional types of attachment pads on a single foot in the Namibia bush cricket Acanthoproctus diadematus (Orthoptera: Tettigoniidae). Proc. R. Soc. B **282**, 20142976. (10.1098/rspb.2014.2976) PMC4590436 26213740

[68] Beutel RG, Gorb SN. 2001 Ultrastructure of attachment specializations of hexapods (Arthropoda): evolutionary patterns inferred from a revised ordinal phylogeny. J. Zool. Syst. Evol. Res. **39**, 177–207. (10.1046/j.1439-0469.2001.00155.x)

[69] Gladun D, Gorb SN. 2007 Insect walking techniques on thin stems. Arthropod Plant Interact. **1**, 77–91. (10.1007/s11829-007-9007-2)

[70] Gorb E, Gorb S. 2002 Attachment ability of the beetle Chrysolina fastuosa on various plant surfaces. Entomol. Exp. Appl. **105**, 13–28. (10.1046/j.1570-7458.2002.01028.x)

[71] Liu Z, Gorb S, Liang H, Bai M, Lu Y. 2024 Leg attachment devices of tiger beetles (Coleoptera, Cicindelidae) and their relationship to their habitat preferences. Insects **15**, 650. (10.3390/insects15090650) 39336618 PMC11432137

[72] Büscher TH, Gorb SN, Eberhard MJB. 2024 Diversity of attachment systems in heelwalkers (Mantophasmatodea) – highly specialized, but uniform. BMC Ecol. Evol. **24**, 130. (10.1186/s12862-024-02319-x) 39455927 PMC11515392

[73] Eberhard MJB, Pass G, Picker MD, Beutel R, Predel R, Gorb SN. 2009 Structure and function of the arolium of Mantophasmatodea (Insecta). J. Morphol. **270**, 1247–1261. (10.1002/jmor.10754) 19434717

[74] Perez Goodwyn P, Peressadko A, Schwarz H, Kastner V, Gorb S. 2006 Material structure, stiffness, and adhesion: why attachment pads of the grasshopper (Tettigonia viridissima) adhere more strongly than those of the locust (Locusta migratoria) (Insecta: Orthoptera). J. Comp. Physiol. **192**, 1233–1243. (10.1007/s00359-006-0156-z) 16868765

[75] Yang H, Engel MS, Shih C, Song F, Zhao Y, Ren D, Gao T. 2023 Independent wing reductions and losses among stick and leaf insects (Phasmatodea), supported by new Cretaceous fossils in amber. BMC Biol. **21**, 210. (10.1186/s12915-023-01720-0) 37807035 PMC10561512

[76] Song H, Amédégnato C, Cigliano MM, Desutter‐Grandcolas L, Heads SW, Huang Y, Otte D, Whiting MF. 2015 300 million years of diversification: elucidating the patterns of orthopteran evolution based on comprehensive taxon and gene sampling. Cladistics **31**, 621–651. (10.1111/cla.12116) 34753270

[77] Jouault C, Nel A, Perrichot V, Legendre F, Condamine FL. 2022 Multiple drivers and lineage-specific insect extinctions during the Permo–Triassic. Nat. Commun. **13**, 7512. (10.1038/s41467-022-35284-4) 36473862 PMC9726944

[78] Shi G, Grimaldi DA, Harlow GE, Wang J, Wang J, Yang M, Lei W, Li Q, Li X. 2012 Age constraint on Burmese amber based on U–Pb dating of zircons. Cretac. Res. **37**, 155–163. (10.1016/j.cretres.2012.03.014)

[79] Wang H, Matzke-Karasz R, Horne DJ, Zhao X, Cao M, Zhang H, Wang B. 2020 Exceptional preservation of reproductive organs and giant sperm in Cretaceous ostracods. Proc. R. Soc. B **287**, 20201661. (10.1098/rspb.2020.1661) PMC7542813 32933445

[80] Schubnel T, Desutter‐Grandcolas L, Legendre F, Prokop J, Mazurier A, Garrouste R, Grandcolas P, Nel A. 2020 To be or not to be: postcubital vein in insects revealed by microtomography. Syst. Entomol. **45**, 327–336. (10.1111/syen.12399)

[81] Limaye A. 2012 Drishti: a volume exploration and presentation tool. In Developments in X-Ray Tomography VIII, SPIE Optical Engineering + Applications, San Diego, CA, USA (ed. SR Stock), p. 85060X. (10.1117/12.935640)

[82] Handlirsch A. 1908 Die fossilen Insekten und die Phylogenie der rezenten Formen; 838 ein Handbuch für Paläontologen und Zoologen [Fossil insects and the phylogeny of recent forms; a handbook for paleontologists and zoologists]. Leipzig, Germany: W. Engelmann. (10.5962/bhl.title.5636)

[83] Huang D, Nel A, Zompro O, Waller A. 2008 Mantophasmatodea now in the Jurassic. Naturwissenschaften **95**, 947–952. (10.1007/s00114-008-0412-x) 18545982

[84] Zhao J., Shih C., Ren D., Zhao Y.. 2011 New primitive fossil earwig from Daohugou, Inner Mongolia, China (Insecta: Dermaptera: Archidermaptera). Acta Geol. Sin. Engl. Edn **85**, 75–80. (10.1111/j.1755-6724.2011.00380.x)

[85] Goloboff PA, Farris JS, Nixon KC. 2008 TNT, a free program for phylogenetic analysis. Cladistics **24**, 774–786. (10.1111/j.1096-0031.2008.00217.x)

[86] Legg DA, Sutton MD, Edgecombe GD. 2013 Arthropod fossil data increase congruence of morphological and molecular phylogenies. Nat. Commun. **4**, 2485. (10.1038/ncomms3485) 24077329

[87] Felsenstein J. 1985 Confidence limits on phylogenies: an approach using the bootstrap. Evolution **39**, 783–791. (10.2307/2408678) 28561359

[88] Nixon CK. 2008 WinClada ver. 1.7. Ithaca, NY: Nixon CK.

[89] Huelsenbeck JP, Ronquist F. 2001 MrBayes: Bayesian inference of phylogenetic trees. Bioinformatics **17**, 754–755. (10.1093/bioinformatics/17.8.754) 11524383

[90] Ronquist F *et al*. 2012 MrBayes 3.2: efficient Bayesian phylogenetic inference and model choice across a large model space. Syst. Biol. **61**, 539–542. (10.1093/sysbio/sys029) 22357727 PMC3329765

[91] Ronquist F, Huelsenbeck JP. 2003 MrBayes 3: Bayesian phylogenetic inference under mixed models. Bioinformatics **19**, 1572–1574. (10.1093/bioinformatics/btg180) 12912839

[92] Lewis PO. 2001 A likelihood approach to estimating phylogeny from discrete morphological character data. Syst. Biol. **50**, 913–925. (10.1080/106351501753462876) 12116640

[93] Rambaut A, Drummond AJ, Xie D, Baele G, Suchard MA. 2018 Posterior summarization in Bayesian phylogenetics using Tracer 1.7. Syst. Biol. **67**, 901–904. (10.1093/sysbio/syy032) 29718447 PMC6101584

[94] Lucas H. 1869 Eurycantha calcarata. Ann. Soc. Entomol. Fr. **4**, 25–26.

[95] Hammer O, Harper DAT, Ryan PD. 2001 PAST: paleontological statistics software package for education and data analysis. Palaeontol. Electron. **4**, 9.

[96] R Core Team. 2023 *R: a language and environment for statistical computing*. Vienna, Austria: R Foundation for Statistical Computing. See https://www.R-project.org/ (accessed 29 October 2024).

[97] Paradis E, Schliep K. 2019 ape 5.0: An environment for modern phylogenetics and evolutionary analyses in R. Bioinformatics **35**, 526–528. (10.1093/bioinformatics/bty633) 30016406

[98] Oksanen J. 2013 vegan: Community ecology package. R package version 2.0-10. See https://cran.r-project.org/package=vegan.

[99] Jaccard P. 1901 Etude de la distribution florale dans une portion des Alpes et du 878 Jura [Study of floral distribution in a portion of the Alps and the Jura.]. Bull. Soc. Vaudoise Sci. Nat. **37**, 547–579. (10.5169/seals-266450)

[100] Peng A. 2025 Supplementary material from: Descending from trees: a Cretaceous winged ice-crawler illuminates the ecological shift and origin of Grylloblattidae. Figshare. (10.6084/m9.figshare.29098256)40527457

[101] Peng A, Engel M, Boderau M, Legendre F, Liu Y, Nyunt TT *et al*. 2025 Supplementary material from: Descending from trees: A Cretaceous winged ice-crawler illuminates the ecological shift and origin of Grylloblattidae. Figshare. (10.6084/m9.figshare.c.7861998)40527457

